# Biological Roles of Resolvins and Related Substances in the Resolution of Pain

**DOI:** 10.1155/2015/830930

**Published:** 2015-08-03

**Authors:** Ji Yeon Lim, Chul-Kyu Park, Sun Wook Hwang

**Affiliations:** ^1^Department of Biomedical Sciences and Department of Physiology, Korea University College of Medicine, Seoul 136-705, Republic of Korea; ^2^Department of Physiology, Graduate School of Medicine, Gachon University, Incheon 406-799, Republic of Korea

## Abstract

Endogenous pain-inhibitory substances have rarely been found. A group of powerful pain suppressor molecules that are endogenously generated are now emerging: resolvins and related compounds including neuroprotectins and maresins. These molecules began to be unveiled in a series of inflammation studies more than a decade ago, rapidly shifting the paradigm that explains the mechanism for the inflammatory phase switch. The resolution phase was considered a passive process as proinflammatory mediators disappeared; it is now understood to be actively drawn by the actions of resolvins. Surprisingly, these substances potently affect the pain state. Although this research area is not fully matured, consistently beneficial outcomes have been observed in a various *in vivo* and *in vitro* pain models. Furthermore, multiple hypotheses on the neuronal and molecular mechanisms for alleviating pain are being tested, deriving inspiration from existing inflammation and pain studies. This paper serves as a brief summary of the proresolving roles of resolvins and related lipid mediators in inflammation and also as a review for accumulated information of their painkilling actions. This also includes potential receptor-mediated mechanisms and discusses future scientific perspectives. Further diverse approaches will help to construct a hidden axis of natural protection principles and establish proofs of concept for pain relief.

## 1. Introduction

Inflammation plays a key role in the pathogenesis of numerous health conditions and is characterized by a series of inflammatory responses. Interestingly, local inflammation promptly occurs after injuries or infections and subsequently involves mechanisms underlying the resolution of inflammation and the wound healing process. Once impaired, these mechanisms may cause chronically harmful conditions. The resolution of inflammation is not simply associated with the reduction of proinflammatory mediators followed by their decreased influences. In fact, different mediators become active for resolving purposes at a certain time point during the inflammatory process, which may define what is currently called the resolution phase. These mediators dynamically stimulate the cellular and biochemical resolution processes that restore the normalcy rather than merely antagonizing the functions of proinflammatory mediators.

Once released due to tissue injuries, proinflammatory mediators induce peripheral pain sensitization; these include bradykinin, prostaglandins, nerve growth factors (NGF), proinflammatory cytokines (e.g., tumor necrosis factor- (TNF-) *α*, interleukin- (IL-) 1*β*, and IL-6) and proinflammatory chemokines (e.g., chemokine (CC-motif) ligand 2 (CCL2)) [1–3]. It is noteworthy that all of these proinflammatory mediators bind to and stimulate specific receptors expressed in the peripheral terminals of nociceptors (pain-mediating sensory nerve fibers) [4]. Once activated, the receptors engage hyperactivation of key transduction molecules, such as transient receptor potential vanilloid subtype 1 (TRPV1) and ankyrin subtype 1 (TRPA1) ion channels, and conduction molecules such as tetrodotoxin-insensitive voltage-gated sodium channels Nav1.7, 1.8, and 1.9. The increases in nociceptor excitability are often more amplified by activation of protein kinases (which is also caused by different downstreams of the same proinflammatory mediator signaling), such as protein kinase A (PKA), protein kinase C (PKC), and mitogen-activated protein kinases (MAPKs) [3–10].

This excessive nociceptor activation initiated in the peripheral terminals is followed by the increased release of neurotransmitters (e.g., glutamate) and neuromodulators (e.g., substance P and brain-derived neurotrophic factor (BDNF)) from the central terminals in the spinal cord [11, 12]. Accordingly, postsynaptic receptors such as N-methyl-D-aspartic acid (NMDA) type glutamate receptors and metabotropic neurokinin-1 receptors are overstimulated, hyperactivating postsynaptic neurons: the central sensitization in the spinal cord dorsal horn [13–15]. Central sensitization plays a key role in the development of chronic pain and even in its persistent spread outside injured areas [16]. The extracellular signal-regulated kinase (ERK), a member of the MAPK family, is specifically phosphorylated in the nociceptive postsynaptic dorsal horn neurons in a hyperactive state. Therefore, phosphorylated ERK (pERK) serves as a marker for central sensitization [17]. In turn, pERK persistently contributes to increasing the activity of NMDA receptors, inhibiting the activity of Kv4.2 potassium channel, inducing AMPA receptor trafficking, and transcribing other pronociceptive genes [14, 17, 18]. Recent studies on a variety of inflammatory responses in the central nervous system (CNS) and postoperative pain have shown that, not only neurons, but also glial cells, such as microglia and astrocytes, play an important role in developing central sensitization of pain [12, 19, 20]. When peripheral tissue is injured, TNF-*α* and IL-1*β* are released from microglia and astrocytes in the spinal cord, where the terminals of the primary nociceptors that cover the injured area form their central synapses [21–23]. This nonneuronal glial activation causes a persistent increase in the pre- and postsynaptic neuronal excitations (e.g., by elevating glutamate and neuropeptide release and by upregulating glutamate receptors) and decreases in the inhibitory mechanisms (e.g., by downregulating inhibitory Cl^−^ signals) in the dorsal horn, thus contributing to central sensitization [21].

In addition, sustained excitation of the cerebral cortex and brain stem on the pain pathway retrogradely (e.g., releasing nitric oxide) facilitates efferent activities of afferent nociceptor neurons in the spinal cord dorsal horn, which also contributes to persistent pain after the onset of tissue injury [16, 24]. Primary nociceptor neurons in a chronic sensitization state also perform efferent activities on injured tissues: the peripheral terminals of those neurons may release substance P and calcitonin gene-related peptide (CGRP) towards the injured site. This may maintain or exacerbate inflammation and widen the inflamed area [25]. Independent of such changes in inflamed areas, the influence of increased synaptic strength and glial activation may extend to neighboring synapses, which can cause a chronic hyperalgesic or allodynic state in a neuronal pathway that covers uninjured areas [26].

TRPV1 and TRPA1 are two critical types of TRP ion channels that participate in pain generation [27]. Besides the influences of proinflammatory cytokines and chemokines which indirectly stimulate TRP channels in a receptor-mediated signal transduction-dependent manner, these two TRPs can also be directly hyperactivated by protons, oxidative stress, tissue stretches, excessively hot or cold temperatures, and proinflammatory lipids such as leukotrienes and isoprostanes, all of which may often occur during inflammation. The consequences from their direct hyperactivations are the same, somatic inflammatory pain* via* central and peripheral sensitization, in which increased glutamate release strongly facilitates the synaptic transmission in the spinal cord [4, 12, 28]. Not surprisingly, sustained TRP activation has also been shown to contribute to chronic pain state. Currently, considerable efforts are being made to develop small-molecule pharmacological inhibitors of these two TRP channels to improve chronic pain [12].

Using unbiased liquid chromatography-mass spectrometry- (LC-MS/MS-) based lipidomics, Dr. Serhan's group at Harvard Medical School uncovered two families of endogenous lipid mediators, including resolvins (e.g., RvD1, RvD2, and RvE1) and protectins (e.g., PD1 or NPD1) in inflammatory exudates [29, 30]. They are biosynthesized from *ω*-3 polyunsaturated fatty acids (PUFAs) such as eicosapentaenoic acid (EPA) and docosahexaenoic acid (DHA). Unlike most known lipid mediators (prostaglandins, leukotrienes, and oxygenated eicosanoids) which are strongly proinflammatory, resolvins and protectins show remarkable resolving potency when tested in animal models of inflammatory diseases [31]. Maresins (e.g., maresin 1), a newly found group of macrophage-derived mediators, are also metabolized from DHA and constitute another strong resolver family during inflammation [32]. Surprisingly, evidence is emerging that resolvins, protectins, and maresins are all able to inhibit the nociceptor activities associated with inflammatory pain [31, 33]. Recent studies have demonstrated that RvE1, RvD1, RvD2, NPD1, and maresin 1 potently suppress somatic inflammatory pain, to some extent by modulating the activities of TRPV1 and TRPA1 and by regulating TRPV1 and TRPA1 activation-elicited synaptic plasticity [28, 33, 34].

In this review paper, we discuss the latest updates on proresolving lipid mediators and attempt to define their roles in the body's natural protective principles, obtain a better understanding of their molecular and cellular mechanisms, and speculate on unanswered questions and therapeutic implications regarding tissue injury-induced inflammatory pain.

## 2. Resolution of Inflammation and Pain

The process by which proinflammatory mediators trigger inflammatory pain is well established as mentioned above. In contrast, little is known about the mechanisms underlying the resolution of inflammatory pain. The hypothesis has often been overlooked that novel anti-inflammatory or proresolving local mediators as well as proinflammatory mediators can naturally occur inside injured tissues. The discovery of such putative resolving molecules would contribute to our understanding of mechanisms for spontaneous or self-limited recovery of acute inflammation and inflammatory pain. Indeed, the resolution of acute inflammation was presumed to be a passive process in the past, but it is now considered to involve homeostatic recruitment of active biochemical programs that return inflamed tissues to their preinflammatory states [12, 42]. Accumulating evidence indicates that anti-inflammation and proresolution are distinct mechanisms that are important for controlling the inflammation [43]. Moreover, proresolving mediators show different actions than known, widely used anti-inflammatory therapeutics such as cyclooxygenase-2 (COX-2) inhibitors [42, 44]. In fact, COX-2 inhibitors are able to disrupt endogenous resolution mechanisms. COX-2 inhibitors reduce not only the production of proinflammatory lipid mediators, but also those of key proresolving lipid mediators, since COX-2 also produces these lipid resolvents. Thus, despite their anti-inflammatory effect, COX-2 inhibitors impair the inflammatory resolution process [30, 42, 44–46]. This is an example. According to an early animal study of pleuritis using rats, the degree of the inflammatory response was decreased at 2 h but was increased even at 48 h after the treatment of NS-398, a COX-2 inhibitor. It was then proposed that COX-2 may have a proinflammatory effect during the early phase of acute inflammation, predominantly seen in polymorphonuclear leukocytes (PMNLs). However, it may also be involved in the resolution of inflammation in the later phase, predominantly seen in mononuclear leukocytes (MNLs) [44]. COX-2 is now known to be required for the biosynthesis of proresolving mediators, such as lipoxins and resolvins, in the resolution phase [42]. This leads to a speculation that effects of COX-2 inhibitors on pain relief should be determined based on the balance between its anti-inflammatory action (or antinociceptive action in pain) and its antiresolving (or pronociceptive) action. It has been suggested that COX-2 inhibitors may transiently exhibit antihyperalgesic effects in some experimental models of chronic inflammation and pain. However, they may also prolong posttreatment pain by disrupting endogenous resolution circuits [47].

The reduction in leukocyte recruitment (influx) to the site of inflammation is indicative of anti-inflammatory responses. Likewise, the increased leukocyte exit (efflux) might be associated with proresolution action [42]. If left unresolved, acute inflammation shifts into chronic inflammation; this transition paradigm has been ignored in the field of pain research. This may be because a lower degree of the inflammation, a feature of local inflammation, seems to represent a chronic state and also it frequently lacks inflammatory signs in the blood indices that are indicative of clear acute inflammation. However, unresolved inflammation can often lead to neuroinflammation that can occur in the CNS and peripheral nervous system (PNS), characterized by the activation of glial cells (e.g., microglia, astrocytes, and satellite glia), which are key contributors to pain exacerbation. Furthermore, persistent chronic inflammation would play an essential role in the maintenance of synaptic plasticity in the neural pain pathway and eventually in chronic pain.

Of note, chronic pain is not an extension of acute pain but originates from plastic changes in pain processing, also known as neuroplasticity [11]. Targeting the transition phase from acute to chronic pain would be critical for prevention of chronic pain that appears to result from chronic inflammation. It should also be noted, however, that it would not be sufficient to treat chronic pain by simply lowering the degree of the inflammation in a chronic phase in the injured tissue because independent chronic mechanisms are already initiated inside the relevant neural circuit in parallel. It is therefore imperative that novel therapies based on “neural” plasticity along the circuit in both the CNS and PNS are developed to control the chronic pain.

## 3. Resolvins

**Figure 1:**
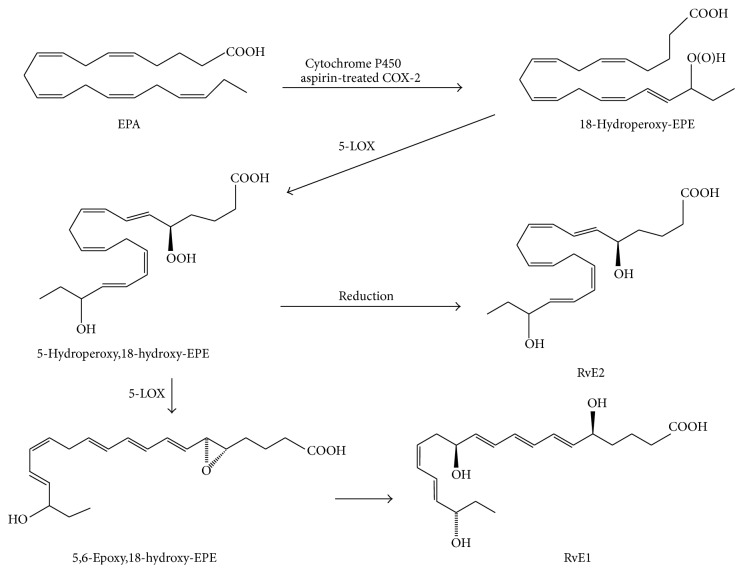
Endogenous biosynthetic pathways of the E-series resolvins (RvEs). Eicosapentaenoic acid (EPA) is converted into 18-hydroperoxy-EPE (18-HpEPE) by aspirin-treated cyclooxygenase-2 (COX-2) or cytochrome P450 (CYP450) and subsequently transformed by 5-lipoxygenase (5-LOX) into RvE1 and RvE2.

**Figure 2:**
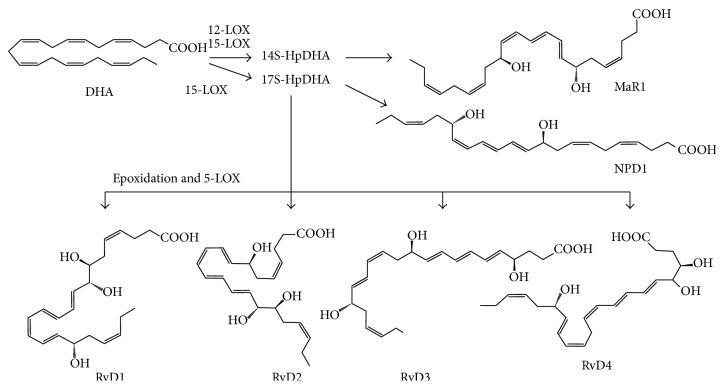
Endogenous biosynthetic pathways of the D-series resolvins (RvDs), maresin 1, and neuroprotectin D1. Docosahexaenoic acid (DHA) is converted into 17S-hydroperoxy-DHA (17S-HpDHA) by 15-LOX. 17S-HpDHA is further transformed into RvD1, RvD2, RvD3, RvD4, or neuroprotectin D1. DHA can be transformed by 12-LOX into 14S-hydroperoxy-DHA (14S-HpDHA), from which subsequent epoxide hydrolysis produces maresin 1.

Resolvins are a new family of local mediators that are present in resolving inflammatory exudates. These are lipids that are enzymatically biosynthesized from PUFAs by multiple types of lipoxygenases, or also by COX-2 as mentioned. Resolvins were initially identified using a systems approach with LC-MS/MS-based lipidomics and informatics. Further research has elucidated the chemical structure and the identities of related intermediate metabolites of resolvins. Resolvins (that were sometimes called resolution-phase interaction products because of their unique nature in biosynthesis: transcellular biosynthesis) are referred to as endogenous compounds synthesized from major *ω*-3 fatty acids, such as EPA and DHA, and are thus denoted as E-series (RvE) and D-series (RvD) resolvins, respectively (Figures 1 and 2) [29]. Like lipoxins, some resolvin members are also produced by the COX-2 pathway in the presence of aspirin, yielding “aspirin-triggered” (AT) forms. Evidence is accumulating that, besides their proresolving mechanisms explained below, resolvins also display potent anti-inflammatory and immunoregulatory effects by inhibiting the syntheses of proinflammatory mediators and modulating the leukocyte trafficking to inflammatory sites as well as clearance of neutrophils from mucosal surfaces.

### 3.1. E-Series Resolvins

Experimental models of inflammation have shown that specialized proresolving lipid mediators (SPMs: a collective term, recently coined for all proresolving lipids) including resolvins have both proresolving and anti-inflammatory actions. Anti-inflammatory actions of resolvins are largely based on downregulation of the recruitment and function of PMNLs and the decreased synthesis and secretion of proinflammatory mediators. For example, they have inhibitory effects against the transendothelial migration of the PMNLs* in vitro* and infiltration* in vivo* [29]. On the other hand, proresolving effects seem to be accomplished by promoting the recruitment and nonphlogistic phagocytosis of monocytes/macrophages and the secretion of IL-10.

The effects of resolvin E1 (RvE1) appear to arise from its specific and direct actions on G-protein coupled receptors (GPRs). RvE1 antagonizes type 1 leukotriene B4 receptor (BLT1) in neutrophils and dendritic cells, and it activates chemokine receptor-like 1 receptors (CMKLR1, also called chemR23) in monocytes. BLT1 antagonism finally suppresses leukocyte infiltration, intracellular signaling of nuclear factor Kappa B (NF*κ*B), and mitogen-activated kinase (MAP kinase), thereby reducing the biosyntheses and secretions of proinflammatory mediators. Strong ChemR23 expression is mostly detected in monocytic cell types, including macrophages, microglia, and dendritic cells. Its activation promotes phagocytic clearance of apoptotic neutrophils. Chemerin is a chemoattractant protein and is a known activator of chemR23. This protein ligand has been shown to exhibit immunological effects similar to those of RvE1 in both* in vivo* and* in vitro* experimental models [48–52]. RvE2 plays a similar role to RvE1, based on receptor-mediated mechanisms for resolving the inflammation [53].

A multitude of experimental inflammation models have been examined to define the resolving outcomes of RvE1 treatment. An experimental model of peritonitis demonstrated RvE1-induced resolution where changes in neutrophil recruitment, dendritic cell migration, and levels of cytokines and chemokines were obvious [54, 55]. Also in the gastrointestinal tract, RvE1 suppressed leukocyte infiltration in animals with colitis. In addition, diverse parameters were explored in the respiratory tract inflammation, and those were again improved upon RvE1 treatment; in an experimental animal model of allergic airway inflammation, the migratory and cytotoxic functions of natural killer (NK) cells were enhanced whereas T helper-17 cells were downregulated. One of the causes seemed to be decreases in levels of proinflammatory cytokines such as IL-23 and IL-6 [56, 57]. Furthermore, RvE1 mitigated antiapoptotic signals and therefore resulted in phagocytosis-induced apoptosis of neutrophils in an experimental model of acute lung inflammation [58]. In a murine model of aspiration pneumonia, neutrophils were less accumulated and the clearance of* Escherichia coli* improved with RvE1 treatment [59].

Topical inflammation was also challenged; in an atopic dermatitis model, proinflammatory signs and indices including inflammatory cytokine levels (IFN-*γ* and IL-4), degrees of CD4(+), CD8(+) T cell infiltration, mast cells and eosinophils, and skin swelling were improved [60]. In a rabbit model of periodontitis, RvE1 resolved local inflammation and attenuated osteoclast-mediated bone loss [61]. Also in a study using human components, RvE1 improved the phagocytic function of macrophages in patients with localized aggressive periodontitis [62]. In a mouse ocular stromal keratitis model infected with herpes simplex virus, there was a significant decrease in the degree of angiogenesis and the frequency and severity of lesions following treatment of RvE1 [63]. RvE1, RvD1, and NPD1 were once shown to display strong effects in protecting neovascularization in a murine model of oxygen-induced retinopathy [49].

### 3.2. D-Series Resolvins

A study with a murine inflammation model using a dorsal-skin air pouch first demonstrated the effects of RvD1 [29]; it interfered with the neutrophil recruitment. Interestingly, despite their similarity in proresolving outcomes, separate GPR types appear to be engaged in the action mechanisms for RvDs and RvEs. That is, RvD1 restricts neutrophil recruitment* via* the activation of lipoxin A4/annexin-A1 receptor/formyl-peptide receptor 2 (ALX/FPR2), which indicates that RvD1 and another SPM lipoxin A4 at least partly share the signaling mechanism for their resolvent actions [64]. RvD1 also seems to utilize a different GPR, GPR32. Recent studies have shown that 17R-RvD1, also known as aspirin-triggered RvD1 and AT-RvD1, activates the same GPRs. However, it remains to be elucidated if the actions of other RvDs involve the same GPR mode [65].

From the molecular actions of RvDs described above one may extrapolate its resolving effects in cellular, tissue, and animal levels. A series of studies on practical inflammation models using experimental animals confirmed it. Like RvEs, RvDs have been shown to be effective in ameliorating inflammatory pathologies of the respiratory and gastrointestinal tracts. RvD1 and AT-RvD1 reversed allergic responses in airway inflammation [66]. RvD1 also dampened inflammation in lipopolysaccharide-induced acute lung injury [67]. In experimental colitis models using dextran sulfate sodium or 2,4,6-trinitrobenzene sulfonic acid, disease parameters were markedly improved upon treatment of D-series resolvins, PMNL infiltration, colonic damage, and body weight loss [68].

Particularly in recent studies using D-series resolvins, the effects in bacterial infection models have been carefully examined. RvD1, RvD5, and NPD1 greatly enhanced bacterial clearance [69]. Furthermore, those resolvin species also elevated the antibiotic effectiveness of bacterial clearance, which leads to a consideration that D-series resolvins may possibly become candidates for novel therapeutics for bacterial infections. For the molecular and cellular mechanisms, RvD5 appears to be the predominant form among endogenous resolvins that work against infection and also utilizes the known RvD1 receptor, GPR32. Downregulations of NF-*κ*B and TNF-*α* expressions upon their treatments were detected. The effect of RvD2 was explored in a sepsis model [70]. RvD2 treatment prevented the induction of sepsis in a murine model of microbial colitis. The mechanism seems to be that RvD2 potently regulates leukocyte function; RvD2 attenuated leukocyte/endothelial interactions* in vivo*, by increasing the production of endothelium-dependent nitric oxide and directly decreasing the expression of leukocyte adhesion receptor. In microbial sepsis initiated by caecal ligation and puncture, RvD2 sharply reduced local and systemic bacterial burden, excessive cytokine production, and neutrophil recruitment. RvD2 also elevated the number of peritoneal mononuclear cells and augmented macrophage phagocytosis. Such multilayered proresolving actions of RvD2 eventually resulted in prolonged survival from sepsis. Taken together, D series resolvins appear to be powerful regulators of both typical and explosive inflammatory responses under infection, by controlling multiple cellular mechanisms mostly aiming to preserve immune vigilance.

Mounting evidence from other disease states and inflamed tissues supports the role of RvDs as potent proresolvents. For example, RvD1 treatment led to less accumulation of apoptotic thymocytes and enhanced the resolution of peritonitis and to wound closing in diabetic mice [71]. Also, RvD1 diminished the production of TNF-*α* and IL-6 in macrophages of the postmortem spinal cord from patients with amyotrophic lateral sclerosis, showing over a thousand times more potency than its precursor lipid, DHA [72]. In the IFN-*γ*/LPS-induced T helper-1 cells of inflamed obese adipose tissues, RvD1 led to reduction of cytokine secretions such as TNF-*α*, IL-6, and monocyte chemotactic protein-1 (MCP-1). Moreover, RvD1 promoted nonphlogistic phagocytosis by macrophages in the stromal vascular fraction. Again, similar effects were obtained from RvD1 and RvD2, both of which reversed the impaired expression and secretion of adiponectin and decreased those of proinflammatory adipokines in inflamed obese adipose tissues, such as IL-6, TNF-*α*, IL-1*β*, and MCP-1 [71, 73]. Those two D-series resolvents also attenuated leukotriene B4-stimulated monocyte adhesion to adipocytes and their transadipose migration.

Further detailed molecular mechanisms for RvD1 actions are now being explored. For example, microRNA- (miRNA-) mediated mechanisms were proposed. In resolving exudates from an experimental model of self-limited murine peritonitis, miR-21, miR-142, miR-146b, miR-203, miR-208a, miR-219, and miR-302d were selectively regulated [74]. Among them, nanomolar RvD1 could upregulate miR-21, miR-146b, and miR-219 and downregulate miR-208a. miR-146b targets NF-*κ*B signaling, and miR-219 targets 5-lipoxygenase, which may cause reduced leukotriene production. Overexpressed miRNA upregulated IL-10 production in macrophages [65]. In ALX/FPR2 knockout mice, RvD1 failed to affect the levels of miR-208 and IL-10 and to decrease leukocyte infiltration, which highlights the GPR specific manner of this mechanism. Toll-like receptor (TLR) involvement was also suggested. RvD1 was detected in mouse kidney under bilateral ischemic insults [75]. RvD1 reduced interstitial fibrosis and leukocyte infiltration and inhibited TLR-mediated macrophage activation, thus contributing to the improvement of kidney morphology and function.

RvD1 also ameliorated oxidative stress-induced inflammation [76]. Oxidative stress finally generates endogenous lipid peroxidation products (LPOs) including 4-hydroxy-2-nonenal (HNE) and 4-oxo-2-nonenal (ONE). In the pain research field, LPOs are known to cause acute pain via direct activation of TRPA1, a major peripheral sensor, but in the inflammation research field, LPOs have been more extensively studied as inflammation inducers that covalently modify protective proteins, for example, glutathione conjugation. RvD1 limited the infiltration of Gr-1(+) leukocytes into the experimentally inflamed region caused by glutathione-conjugated HNE injection.

## 4. Effects of Resolvins on Inflammatory Pain

D and E series resolvins are now known to powerfully lead tissue situation to the resolving phase in a variety of forms of inflammation. By this notion, a hypothesis can be born that pain, an important aspect of inflammation (so-called “dolor” in* Latin*), may be strongly affected by the actions of resolvins. At a different point of view, resolvins may also directly regulate the function of the sensory neural circuit, independent of their control mechanisms for the inflammatory state; in fact unsaturated nature and lipoxygenase-involved biosynthetic pathways closely resemble a group of lipidergic mediators that directly act on the nerve [77, 78]. No matter what mechanism explains the action of resolvins on pain, a large number of inflammatory pain models have been tested to determine whether resolvins affect pain states, and as a result surprising analgesic effects have consistently been reported [30, 31, 79] (Table 1). A pioneering result from the study by the Ji lab stands at the forefront of the resolvin-pain research [35]. This study focused on inflammatory pain and commonly on multiple types of experimental animal models such as inflamed animals using complete Freund's adjuvant (CFA), carrageenan, and TNF-*α* and on an acute pain model using capsaicin, a pain receptor TRPV1-specific agonist; RvE1 administration drastically decreased pain symptoms even at nanogram levels.

**Figure 3:**
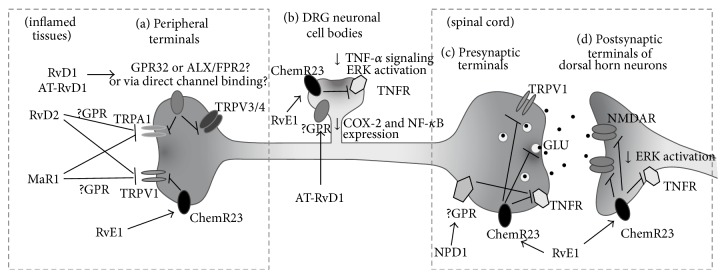
Signaling mechanisms of resolvin actions on the pain-mediating DRG neurons and their synapses. (a) RvE1 suppresses TRPV1 activity, and RvD1 suppresses TRPA1, TRPV3, and TRPV4 activities in DRG nerve terminals. (b) RvE1 attenuates TNF-*α* signaling and ERK activation in DRG neuronal cell bodies. In the same regions, AT-RvD1 downregulates COX-2 and NF-*κ*B expressions. (c) RvE1 reduces excitatory neurotransmitter releases and TNF-*α* signaling at the central presynaptic terminals. (d) RvE1 suppresses overactivated ERK and NMDA receptor at postsynaptic dorsal horn neurons in the spinal cord. DRG, dorsal root ganglion; GLU, glutamate; NMDAR, NMDA receptor; TNFR, TNF-*α* receptor; TRPA1, transient receptor potential ankyrin subtype 1; TRPV1, transient receptor potential vanilloid subtype 1; TRPV3, transient receptor potential vanilloid subtype 3; TRPV4, transient receptor potential vanilloid subtype 4.

The underlying mechanisms that realize such powerful analgesic outcomes might be complex and still require further elucidation, but clear evidence for some downstream signalings was provided. First, a typical mechanism as shown above in the inflammation research field also works in the inflamed tissues of pain models. When intraplantar injections were given into inflamed paws, RvE1 disturbed neutrophil infiltration and paw edema formation and reduced expression of proinflammatory cytokines and chemokines such as IL-1*β*, IL-6, TNF-*α*, MCP-1, and macrophage inflammatory protein-1*α* (MIP-1*α*) [35]. Another mechanism is via direct actions on the neuronal circuit (Figure 3). RvE1 has been shown to exert direct effects on the primary nociceptor neurons. TRPV1 is a major pain receptor present in the tissue-innervating terminals of nociceptor neurons, detecting and electrically transducing damage signals including heat, anisotonicity, acid, and capsaicin, in normal and inflammatory conditions. Thus, TRPV1 has been a key peripheral analgesic target for nearly two decades. RvE1 blocked capsaicin-evoked acute pain and TRPV1-mediated heat pain. Its detailed molecular mechanisms presumably merge into the GPR signaling mentioned above, G*α*i signaling- (which is GPR's intracellular downstream signaling-) mediated modification of TRP functions [28, 35]. ChemR23 GPR is expressed in the dorsal root ganglia where nociceptor neuronal cell bodies are clustered. Its colocalization with TRPV1 in DRG neurons has already been demonstrated [35].

This GPR-mediated molecular mechanism may work not only in the peripheral part of the TRPV1-positive nerve circuit, but also in the central synapses. This is because the same signaling machinery is preserved in both pre- and postsynapses in the dorsal horn of the spinal cord and even in the adjacent microglia that may support synaptic strength. It can therefore be inferred that similar pain-inhibitory mechanisms might be involved when RvE1 is intrathecally injected or endogenously generated. However, the final postsynaptic effectors that lead to pain alleviation should be different from those present in the peripheral terminals or central presynaptic terminals of the DRG nociceptor population. The peripheral or presynaptic effectors of the nociceptors are TRP channels as mentioned, while the central postsynaptic effectors expressed in dorsal horn neurons appear to be NMDA receptors. On the other hand, both mechanisms seem to share extracellular signal-regulated kinase (ERK) inhibition as an intermediate downstream process [80, 81].

The microglia located in the spinal cord dorsal horn play a critical role in maintaining pathologic plasticity in the nociceptive synapses between DRG and dorsal horn neurons. The monocytic origin of spinal microglia leads to a strong possibility that the microglia may transcellularly synthesize and secrete resolvins and also express the resolvin-specific GPRs, as already shown in the monocytes and macrophages [41]. Further research may investigate the quantity and type of resolvins that are secreted from microglia upon injury or inflammation and how much the GPRs contribute to the analgesic effects when extraneous resolvins are administered in pain synapses.

It is also noteworthy that intrathecal RvE1 had a greater efficacy in dampening the second pain phase in formalin-induced models, as compared with NS-398, a COX-2 inhibitor, and even with morphine, the famous opioid. Furthermore, the analgesia seems to follow an indiscriminate manner, for example, when administered intrathecally, RvE1 or RvD1 alleviates both heat and mechanical hypersensitivity. In the same experimental models of pain, chemerin, a ChemR23-specific GPR ligand, also exhibited similar effects. Such GPR signalings seem to be a predominant mechanism underlying the analgesic effects of resolvins on the neuronal circuit in multiple types of pain, which is based on the series of Ji group's experiments [35]. Particularly in the central mechanism underlying the modification of synaptic strength, this GPR-dependent aspect appears to be obvious. In the second pain phase of the formalin-induced pain model, the analgesic effects of RvE1 were blocked by the intrathecally injected G*α*i-coupled GPR inhibitor (pertussis toxin) and were also inhibited when spinal ChemR23 was genetically knocked down. In addition, as mentioned above, chemerin successfully replicated the intrathecal effects of resolvins. However, little is known about the relationships between the inhibitory effects of D series resolvins and GPR32 or ALX/FPR2 for painkilling mechanisms in the spinal synapses. It has once been reported that ALX/FPR2 is expressed in spinal astrocytes [82].

For resolvins' analgesic actions, not only the mechanism in the spinal synapses, but also the peripheral mechanisms seem to depend on the GPR signalings. In DRG neurons, RvD2 inhibited both of activities of TRPV1 and TRPA1 (Table 2) [34]. The effect of RvD2 was prevented by the pretreatment of pertussis toxin or GDP*β*S which is able to block G*α*i-coupled signaling. This suggests that the TRP channel blockade is mediated by GPR action. However, identities of RvD2-specific GPRs remain obscure. Interestingly, these unknown GPR to G*α*i-cascades are likely coupled only to specific subtypes of TRP channels. Accordingly, pain-inhibitory effects of resolvins might be limited to the sensory modalities (e.g., heat, cold, pressure, and poking) that the target TRP channel conveys. That also seems to be true for RvE1, since RvE1 applications resulted in clear inhibition of TRPV1 activity, but not of TRPA1 [34, 35].

TRP channel specificity of resolvins' actions was further explored by the Hwang lab. Unlike the sharp specificity of RvE1 to TRPV1 activity, RvD1 displayed broader inhibitory actions on the activities of TRPA1, TRPV3, and TRPV4 [36, 39]. Activities of other nociceptive sensory TRP channels including TRPV1, TRPV2, and TRPM8 were inert to RvD1 applications. Different TRP channels are known to be in charge of different pain phenotypes according to their sensory modalities. That is, TRPA1 and TRPV4 are mechanosensitive channels and thus mediate mechanical pain, and TRPV3 and TRPV4 are heat-gated channels and therefore contribute to heat pain. TRPV4, which is also activated by hypotonicity, is responsible for hypotonicity-induced pain. Each individual TRP also mediates acute pain induced by its specific chemical agonists such as cinnamaldehyde or formalin for TRPA1 and camphor for TRPV3. Therefore,* in vivo* specificities of RvD1 on such particular pain phenotypes could be presumed from the* in vitro* TRP channel results. Predictably, the peripheral treatment of RvD1 prevented agonist-specific acute pain. TRPA1 and TRPV4 mediate mechanical hyperalgesia and allodynia under complete Freund's adjuvant- (CFA-) induced inflammation, which is explained by their sensory modalities as mentioned above. Inflammatory pain was also prevented by RvD1 injections. Hypotonicity-induced pain (which is presumably mediated via TRPV4 activation) of animal paws primed with an inflammatory mediator prostaglandin E2 was also suppressed by local RvD1 treatment. Heat hyperalgesia (which TRPV3 and TRPV4 activations contribute to) in CFA-induced inflammation was also reversed. Formalin is a standard substance that causes pain in examining the peripheral (phase 1) and central (phase 2) mechanisms of an analgesic compound [83]. The phase 1 mechanism is based on acute nociception* via* TRPA1-direct activation by formalin, and RvD1 significantly blocked this phase. Collectively, broad specificity of RvD1 to pain receptor TRP channels appears to explain such an extended analgesic spectrum for variable pain phenotypes. More importantly, pain alleviation by extraneous resolvin injections at least in the acute phase can be independent of a typical inflammatory mechanism where leukocytes should be key players.

AT-RvD1 has shown different specificity; it only has an inhibitory effect on TRPV3. This was consistent with behavioral phenotypes seen following the use of TRPV3 agonists. In addition, AT-RvD1 was also effective at inflammatory heat responses (which is TRPV3's physical modality) triggered by CFA or carrageenan injections [39]. However, no changes in mechanical phenotypes were detected at least immediately after the peripheral application of AT-RvD1. This time course is, again, in an earlier phase than leukocyte-mediated resolving mechanism begins. Different from the GPR-mediated interaction between RvE1 and TRPV1, a distinctive mode may work for interactions between AT-RvD1 and TRPV3 [39]. For example, no inhibitory effect was replicated with the treatment of different agonists for the same RvD receptor (ALX/FPR2), such as cathelicidin LL-37 and Trp-Lys-Tyr-Met-Val-Met [36]. In addition, the inhibitory effects of AT-RvD1 against TRPV3 were maintained following treatment of gallein (a G*βγ* inhibitor that incapacitates GPR signalings). These results suggest that it is not likely that inhibitory effects of RvDs depend on GPR signaling. Furthermore, the potency of RvD actions on TRP channels is different from the known potency of RvD actions on leukocytes that likely involve GPR signaling, indicating that different receptors with different affinities are engaged [84].

Many PUFAs and their metabolites have been demonstrated to directly bind to and modulate TRP channels. Resolvins seem to share the biosynthetic pathways and structural features including carbon chain length and number of double bonds with known TRP channel binding lipids, which stimulates the curiosity whether resolvin inhibits channel gating by direct TRP binding. Further studies, such as binding assays, mutation studies on the putative binding sequences, or even deorphanizing hidden target GPRs, need to precede the conclusion. The Ji lab and the Hwang lab have utilized different resolvins for their molecular mechanism studies. The GPR-mediated mechanism and the TRP-specific mechanism might be differentially important for each tested resolvin. Further, either of the molecularly different mechanisms may be differentially available, or their contribution indices may vary depending on cell types or pathologic conditions.

No matter what the predominant intracellular and molecular mechanism is, the above independent results from neurons and neural circuits clearly verified that neuronal cells and microglia are important targets for resolvin actions. Moreover, outcomes from all these actions are surprisingly consistent and those aim to fight pathologic pain and few adverse effects are predicted. In addition, both Ji and Hwang labs have shown that resolvins did not alter thresholds for normal pain despite their high efficacy and potency [34–36, 39]. To verify this, Ji et al. further measured the strength of the central synapse and no change in the basal spontaneous excitability of postsynapses was detected after RvE1 treatment. This indicates that it might be more appropriate explanation that resolvins can “normalize” neuronal function rather than simply “downregulating” it when synaptic strength is exaggerated due to pathologic inflammation [34, 35].

## 5. Neuroprotectin D1

In 15-lipoxygenase metabolism, DHA generates D series resolvins and NPD1 [85, 86]. The term “protectin” is based on its anti-inflammatory and protective actions in the nervous system. To describe their origin more specifically, the prefix “neuro-” is sometimes added, as in “neuro-” protectin D1 (NPD1) but NPD1 is detected in other nonneuronal regions including the lungs and blood [87]. Like resolvins, NPD1 exerts inhibitory effects on PMNL infiltration [88]. In the nervous system, NPD1 seems to be produced mostly by glial cells and also acts on them in an autocrine or paracrine manner, reducing cytokine expression. Multiple murine experimental models have shown that NPD1 ameliorated damaging pathology in corneal injuries, oxidative-stress-induced retinal epithelial pigmentation, and brain cells exposed to *β*-amyloid. It also reduced stroke occurrence and promoted wound healing in the cornea [6, 89, 90]. Regardless of its origin, when extraneously administered, NPD1 is also able to display protective effects. In a peritonitis model using zymosan A, the effects of nanogram NPD1 injections were examined in terms of leukocyte infiltration. Collected peritoneal lavages at 4 h showed a greater than 90% reduction in PMNL infiltrations, which indicates that their migration and infiltration were powerfully suppressed* in vivo* [91]. An interesting result was obtained from a murine peritonitis study using NPD1 and RvE1. Separate injections of nanomolar NPD1 and RvE1 led to significant inhibitions of PMNL infiltrations and the degree of NPD1 effect was lower than that with RvE1. When administered concomitantly, greater inhibitory effects were detected on the infiltration of PMNLs, indicating that an* in vivo* additive outcome can be acquired using coapplication of NPD1 and RvE1 [87, 92]. Observations of NPD1 occurrence and function in nonneuronal tissues become active. In the immune system, eosinophils were demonstrated to be able to produce NPD1 during the resolution of inflammation [93]. NPD1 is also generated in murine joints during the resolution of Lyme disease [94]. NPD1 exhibited renoprotective effects in ischemic renal injury models [95]. The production of adipokines could be regulated by NPD1 in an obesity model [96]. Stem cell differentiation is also affected; the incubation in nanomolar NPD1-containing media greatly promoted neuronal differentiation whereas leukotrienes lacked this effect [97].

Eventually, Park et al. demonstrated that NPD1 is also a highly potent endogenous inhibitor of TPRV1, a negative regulator for spinal cord synaptic transmission, and consequently a suppressor of inflammatory pain [28]; NPD1 blocked TRPV1- and TNF-*α*-mediated synaptic transmission and long-term potentiation (LTP) in the spinal cord. NPD1 blocked capsaicin-induced TRPV1 currents in DRG sensory neurons at very low concentrations. The IC_50_ of NPD1 (0.36 nM) for TRPV1 inhibition was ~500 times lower than that of AMG9810, a widely used synthetic TRPV1 antagonist. NPD1's action on TRPV1 is likely mediated by activation of specific pertussis toxin-sensitive/G*α*i-coupled GPCRs and subsequent inhibition of adenylyl cyclase, PKA, and ERK signaling pathways. Low dose spinal injection of NPD1 (0.1–10 ng) prevented spinal long-term potentiation of the synaptic transmission and reduced TRPV1-dependent inflammatory pain, again, without affecting baseline pain. NPD1 also reduced TRPV1-independent but TNF-*α*-dependent pain hypersensitivity.

## 6. Maresins: Macrophage-Derived Resolvents for Inflammation and Pain

Macrophages play a central role in maintaining homeostasis against injuries, infections, and diseases by orchestrating resolution and tissue repair [98]. Correspondingly, macrophages utilize (secrete and respond to) resolving lipid mediators as mentioned above for accomplishing their purposes and also participate in transcellular biosynthesis of those lipids. Besides known lipid resolvents found so far, the presence of other mediators is still possible regarding the extended influences of this cell type on our body protection and the detection of undesignated lipid intermediates the roles and origins of which still remain obscure. Consequently, attempts to identify a novel lipid mediator generated from macrophages and challenges to define its actions and biosynthesis led to the discovery of a novel group of DHA-derived resolvents, maresins [99].

In a lipidomics study using self-resolving inflammatory exudates from a murine model of peritonitis, accumulations of 17S-hydroxyDHA (17S-HDHA), 14S-HDHA were detected [29, 99]. 17S-HDHA is known to be a marker for the biosynthesis of D-series resolvins and NPD1 since it is an upstream intermediate. On the other hand, 14S-HDHA was not known for its further metabolism. It could be hypothesized that 14S-HDHA might be further metabolized to serve as a marker on a novel biosynthetic pathway for a certain bioactive final product. Incubating macrophages with either DHA or 14S-hydroperoxyDHA (14S-HpDHA, the immediate precursor metabolite for 14S-HDHA) resulted in an occurrence of a converted form with highly potent anti-inflammatory and proresolving actions. A series of experiments such as isotope incorporation, intermediate trapping, and characterizations of physical and biological properties showed that the macrophage-produced mediator is 7R,14S-dihydroxy-4Z,8E,10E,12Z,16Z,19Z-DHA, which is synthesized* via* a novel 12-lipoxygenase action. This substance was termed as maresin 1 (macrophage mediators in resolving inflammation 1 (MaR1) [99]; recently, MaR2 (13R,14S-diHDHA) was found [100]). As briefly mentioned above, maresins exert potent anti-inflammatory and proresolving actions. Cellular mechanisms appear to be similar to other resolvents. MaR1 inhibits the infiltration of PMNLs and promotes the macrophage phagocytosis of apoptotic cells.

MaR1 also has recently been shown to exert potent analgesic actions. Intraplantarly administrations of MaR1 alleviated mechanical allodynia in a neuropathic pain model in mice mimicking chemotherapy-induced pain, by injecting vincristine intraperitoneally. MaR1 also attenuated capsaicin- or allyl isothiocyanate- (a TRPA1 agonist-) induced acute pain, which suggests that MaR1 may modulate TRPV1 and TRPA1 directly or indirectly. Indeed, MaR1 suppressed TRPV1 activity in cultured neurons of DRG [32]. MaR1 blocked capsaicin- (100 nM) induced inward currents (IC_50_: 0.49 ± 0.02 nM) in a dose-dependent manner, which was reversed by pertussis toxin treatment, indicating a GPR-mediated TRPV1-inhibitory mechanism. However,* in vitro* TRPA1 activity was resistant to MaR1 application, suggesting an unknown indirect mechanism for the effects of MaR1 on allyl isothiocyanate-evoked pain and mechanical allodynia.

Collectively, resolvin series substances have consistently been confirmed to possess significant pain resolving effects and the related substances, NPD1 and maresins, are emerging in the research field in the same context.

## 7. Conclusions and Perspectives

This review summarizes information on resolvins and related substances, which are important both as endogenous proresolvers and as therapeutic candidates preventing deterioration of inflammation and pathologic pain. Indeed, clinical applications related to inflammation are seriously being considered; a clinical phase II trial of a resolvin-derived synthetic analog RX-10045 for eye dryness was recently completed. A phase I study of oral administration of RvE1 was also completed, and its possible clinical applications will likely include rheumatoid arthritis, asthma, and colitis. Studies on the total synthesis processes of resolvins will help to establish a platform to generate more chemically and metabolically stable analogs which guarantees sufficient proresolving duration when clinically applied [101]. Resolvin studies, as mentioned above, are also evolving into ones investigating practical utilities for various pain diseases. In the initial stage, a relatively stable analog already began to be examined regarding painkilling efficacy [35].

Although the powerful potencies and negligible adverse effects of resolvins appear to promote their transitions to clinical settings, many aspects of the biological mechanisms remain to be clarified. Early termination of inflammation by resolvin treatment seems to be beneficial in that it prevents conversion into chronic inflammation and chronic inflammatory pain. On the other hand, if it is a premature termination, clearance of initial damaging insults or microorganisms might be incomplete. Surprisingly beneficial indices were obtained in this regards; RvDs and RvE1 contribute to bacterial clearance [59, 69]. In addition, resolvins appear to promote injury repair by elevating phosphoinositide 3 kinase-dependent migration and reducing apoptotic cell accumulation [102, 103].

Although resolvins are found in inflammatory exudates, which is the reason we call these endogenous substances, most studies about the effects and mode of actions have been conducted using exogenous administration. Quantitative information on the tissue-level oscillations needs to be more accumulated. In the same context, it remains to be clarified which type of cells (or blood albumin) is the major source for their metabolic precursors, DHA and EPA [104]. During transcellular synthesis, rate-limiting cell types for resolvin production have yet to be identified. In pain-mediating neural circuit research, such observations on the endogenous regulation might be more complex because, particularly near the central synapses, changes in the leukocyte content are limited. Changes in functional parameters (resolvin production, receptor expression, receptor sensitivities, etc.) of neural components, but not cell numbers, might become dynamic and critical for relaying resolving signals unlike peripheral tissues. Possibility of the presence of other unknown target receptors or ion channels should be also speculated. In addition, the potencies of other lipid mediators that are not classified as resolvin species but share biosynthetic pathways need to be carefully compared for resolution and anti-inflammation [105].

Taken together, recent efforts initiated the definition of the powerful proresolving and analgesic actions of resolvins and related lipidergic molecules. Studies are now examining their cellular and molecular mechanisms. Further studies will explore and examine the utilities for more specific types of pain diseases including mostly chronic ones and will also expand the information to explain an axis of the body's natural protective principles.

## Figures and Tables

**Table 1:** Summary of important antinociceptive actions of resolvins in various pain models.

Resolvins		Genus	Pain models	Pain symptoms that were improved by resolvin treatment [references]
D-series	RvD1	Mouse	CFA	Mechanical allodynia, heat hyperalgesia [35, 36], mechanical hyperalgesia [36]
			Carrageenan	Chemical pain [36]
			Formalin	Phase 1 and 2 inflammatory pain [35, 36]
			Prostaglandin E2	Acute mechanical pain [36]
			TRP agonists	Acute chemical pain [36]
			Incision	Mechanical allodynia [35]
		Rat	Incision	Mechanical allodynia and hyperalgesia [37]
			Trinitrobenzene sulfonic acid-induced chronic pancreatitis	Mechanical allodynia [38]
	RvD2	Mouse	CFA Carrageenan	Mechanical allodynia and heat hyperalgesia [34]
			Formalin	Phase 2 inflammatory pain [34]
	AT-RvD1	Mouse	CFA	Heat hyperalgesia [39]
			Carrageenan	Chemical pain [39]
			TRP agonists	Heat hypersensitivity [39]
		Rat	Carrageenan	Mechanical allodynia [40]

E-series	RvE1	Mouse	CFA Carrageenan Intrathecal TNF-*α*	Mechanical allodynia and heat hyperalgesia [35]
			Formalin	Phase 1 and 2 inflammatory pain [35]
			TRP agonists	Acute capsaicin-evoked pain [35]
			Spinal nerve ligation	Heat hyperalgesia [35]
			Chronic constriction injury	Mechanical allodynia and heat hyperalgesia [41]

Others	NPD1	Mouse	CFA	Heat hyperalgesia [28]
			Formalin	Phase 2 inflammatory pain [28]
			TNF-*α*	Mechanical allodynia and heat hyperalgesia [28]
			TRP agonists	Acute capsaicin-evoked pain [28]
	Maresin 1	Mouse	TRP agonists	Acute capsaicin-evoked pain [32]
			Vincristine chemotherapy	Mechanical allodynia [32]

**Table 2:** IC_50_ (nM) values for the inhibition of TRPV1 or TRPA1 activity by resolvins, maresin 1, NPD1, and their fatty acid precursors as obtained from studies using DRG neurons. Capsaicin (100 nM) and allyl isothiocyanate (300 mM) were used as basal agonists for activation of TRPV1- and TRPA1-mediated currents, respectively.

Inhibitors	TRPV1 IC_50_ (nM)	TRPA1 IC_50_ (nM)
RvE1	1 ± 0.14	—
RvD1	—	8.5 ± 0.13
RvD2	0.09 ± 0.02	2.1 ± 0.53
DHA	1200 ± 20	—
EPA	224 ± 10	—
MaR1	0.16 ± 0.01	—
NPD1	0.36 ± 0.05	—
